# Considering context in area-level socioeconomic status, cancer treatment initiation, and survival

**DOI:** 10.1093/jncics/pkad078

**Published:** 2023-10-17

**Authors:** Matthew F Hudson, Alicia M Oostdyk, Virginia M Simmons, Julie C Martin

**Affiliations:** Department of Medicine, Prisma Health Cancer Institute, Greenville, SC, USA; Department of Medicine, Prisma Health Cancer Institute, Greenville, SC, USA; Department of Medicine, Prisma Health Cancer Institute, Greenville, SC, USA; Department of Medicine, Prisma Health Cancer Institute, Greenville, SC, USA

In this issue of the Journal, Guadamuz et al. (1) consider socioeconomic status as a hierarchical social structure predicated upon wealth, prestige, and power; the authors further conceptualize socioeconomic status by income, wealth, education, occupation, and living conditions. Their work further considers the relationship between area-level socioeconomic status and treatment initiation and survival among patients with cancer by using census block estimates of median income, home values, rental costs, poverty, blue-collar employment, unemployment, and education information. Investigators observed that lower area-level socioeconomic status was associated with lower treatment rates and worse survival among patients with cancer inequities that persisted amidst the COVID-19 pandemic.

Cancer care delivery research typically aspires to execute intervention studies targeting modifiable clinical and practice-level factors in the care delivery environment (2), but Guadamuz et al.’s (1) “area level” considerations exemplify cancer clinicians’ increasing attention to social determinants of health (3,4)—that is, economic stability, access to quality education, health-care access and quality, neighborhood and built environment, and social and community context. Social determinants, generally, are related to carcinogenic exposure (5), cancer screening (6), cancer care (7,8), and cancer prognosis (9) and persistent related disparities (10). Thus, cancer care clinicians and researchers welcome research tools such as the Yost Index (11) that the authors employ to calculate area-level socioeconomic status or the Area Deprivation Index (12,13), which quantifies relationships between neighborhood (technically, zip code tabulation areas) and health outcomes (14). These measures may encourage even deeper consideration of area-level correlates beyond liquidity. The findings of Guadamuz et al. may encourage our field to consider the built environment and community resources (15) that may more precisely explain the relationship between area-level socioeconomic status and cancer treatment and survival.

The built environment (eg, land use and housing) may particularly predict chronic disease (16), with housing especially relevant to cancer (17). Indeed, land use and housing’s mere outward appearance may more precisely explain cancer care–related behavior and impact. Psychologist Philip Zimbardo’s late 1960s and early 1970s inquiry into vandalism observed that slightly compromised objects (in his study, an ostensibly inoperable automobile) left unattended in a neighborhood encouraged destructive behavior, portending a disordered environment (18,19). This work later encouraged Wilson and Kelling (20) to propose the “broken window” theory: Neighborhood artifacts (eg, windows, lampposts, houses) left unrepaired would result in more or all artifacts damaged, ultimately creating a climate of social disorder. Wilson and Kelling also posited that neighborhood income does not protect against this phenomenon (ie, higher–socioeconomic status neighborhoods will also succumb to social disorder amidst unaddressed damage). Later, Cohen and colleagues (21) observed that a “broken window” index explained more variance in gonorrhea rates than a poverty index, including income, unemployment, and low education. Cohen et al. proposed that the disordered area potentially acts as a cue, indicating that the environment accepts, condones, or encourages risky behavior and predicting gonorrhea. The broken window theory’s relevance to communicable disease may encourage cancer care delivery researchers to ponder its relevance to cancer care delivery and impact. O’Brien and colleagues (22), however, observed that although the broken window theory is potentially correlated with mental health, substance use, and overall health, there is no consistent evidence for its relationship to physical health or risky behavior. Also, broken window–motivated efforts to “clean up” the environment or restore order may adversely affect the marginalized community the effort proposes to benefit. Kamalu and Onyeozili (23) observed that broken window theory–motivated law enforcement resulted in disproportionate arrests of non-Hispanic Black and Hispanic youths. Kamalu and Onyeozili proposed that other social conditions, such as unemployment, education, and drug use, may more accurately predict community wellness. Cancer care delivery researchers, then, should further debate the broken window theory’s merits relative to cancer care delivery outcomes and survivorship disparities. Qualifications notwithstanding, the broken window’s meso-level perspective guides cancer care delivery research toward community-based drivers (or suppressors) of cancer preventive or management artifacts embedded in low area-level socioeconomic status locales.

Earlier research indicated that profit and affluence predict for-profit hospitals’ locations, thus intentionally marginalizing Medicaid and uninsured patients (24,25). More recent studies suggest an artifact or persisting inclination: rural non-Hispanic Black individuals travel longer distances to hospitals than rural White individuals (26). Thus, the geographic location of care facilities potentially deprives historically disenfranchised populations of primary care—an enterprise favorably affecting cancer prevention and decreased mortality (27). These previous observations support Guadamuz et al.’s (1) admonition to encourage federal interventions addressing persistent under-investment in healthcare systems serving historically marginalized areas. Apart from geography, care advocates understand that historic and current insurance restrictions disproportionately compromise minority populations (28); Guadamuz et al. encourage revisiting point-of-care improvements (eg, reimbursement for navigation services) that may create financial incentives for care equity. Guadamuz et al.’s suggestions align with Dahlgren and Whitehead’s (29) socioecologically informed call for policy development at multiple levels: level 1, economic strategies and tax policies begetting structural changes; level 2, enhanced health services that target living and working conditions; level 3, strengthening social support; and level 4, influence of individual lifestyles and attitudes.

Multilevel policies may beget care access benefitting cancer prevention and survivorship, but enhanced facility proximity and care reimbursement, although necessary, is likely insufficient for optimizing care for patients residing in low area-level socioeconomic status locales. Health-care organizations and their care teams may create and sustain care equity threats. Workforce conditions, such as unfavorable patient to clinician ratios (30) potentially produce adverse patient outcomes and reduce clinician health (31). Suboptimal working conditions may encourage actions that circumvent or temporarily fix real or perceived workflow hinderances or compromised system design flows to provide care (32). Often, systems value these “workarounds” as examples of clinicians’ dedication to overcoming the impediments they encounter (32), but workarounds can create short-term hazardous circumstances for patients. Overreliance on overcoming rather than addressing underlying problems potentially normalizes substandard care and risky behavior. These workarounds, then, potentially increase patient exposure to adverse events and preventable harm. Churruca and colleagues (33) appropriated the aforementioned broken window theory to health-care organizations determining whether disorder—exemplified by practice standard violations (eg, taking short cuts in reading a drug dose or in washing hands)—may exemplify a “social disorder” that portends suboptimal care quality and safety. Churruca et al. feared that as staff increasingly tolerate, normalize, and even require deviations from practice standards, the environment permits a “normal illegal” (34)—that is, sanctioned transgression of practice standards and guidelines. Researchers more deeply considering Guadamuz et al.’s findings may examine whether care organizations exhibiting these characteristics disproportionately reside in lower area-level socioeconomic status locales.

We encourage hypothesis testing that considers relationships between the built environment, practice norms (potential area-level socioeconomic status attributes), and cancer treatment initiation and survival. How might we design studies to test these relationships? Cancer care delivery espouses advancing beyond traditional randomized clinical trial paradigms (prioritizing internal validity) to embrace pragmatic trial methods that prioritize external validity and the relevance of community practice (35). Moreover, cancer care delivery research embraces cluster-randomized trials that assign sites (not patients) to conditions; this approach accounts for the contextual influence of “place”—typically, clinics or health-care facilities—having a global impact on patient care and outcomes (36). This perspective implicitly concedes that “where” stakeholders implement an intervention potentially informs “how” the intervention functions and ultimately affects the patient. Guadamuz et al.’s findings encourage an even broader exploration beyond traditional care confines to understand how people’s birthplace as well as how and where they, grow, work, live, and age moderates or mediates an intervention’s impact (37). Articulating area-level socioeconomic status attributes, such as the broken window index, helps us understand how social determinants “moderate” (ie, affects the direction and strength of) the relationship between clinical care and cancer care outcomes. These same social determinants may also mediate (ie, explain the “how” or “why” of) care delivery outcomes. Many researchers (38-40) assert that cancer care delivery research requires examining multiple levels of influence in addition to patient, family, and clinician factors. Taplin et al. (38) identified organization level, community environment, state policy, and federal policy as opportunity-rich domains for research. Further, Taplin et al. encouraged us to develop interventions that address the patient and at least 2 levels of contextual influence, thereby targeting at least 3 levels of influence. Guadamuz et al.’s findings provide an opportunity to expand this thinking beyond the clinician and clinic to examine the characteristics of the host community and the organizational context of care facilities serving low area-level socioeconomic status locales. Cancer care delivery researchers eschewing this consideration may continue “looking under the lamppost,” examining care delivery facilities or activities exhibiting the greatest expenditures but potentially ignoring larger public health–based opportunities that more comprehensively improve cancer survivorship (41).

Guadamuz et al. (1) reveal an area-level socioeconomic status relationship with cancer treatment initiation and survival among people with cancer. These findings align with the field’s growing attention to social determinants of health. These findings may encourage cancer care delivery researchers to expand their inquiry beyond the clinical practice domain to include potential underlying socioeconomic status attributes (eg, the state of the built environment, organizational resources and practices) embedded in low area-level socioeconomic status locales. We encourage a multilevel research perspective to inform hypothesis generation and testing that may clarify the relationships Guadamuz et al. observe. Ultimately, these insights will inform more precise meso-level and macro-level interventions that either mitigate adverse attributes embedded in low area-level socioeconomic status locales or supplement the sound and healthy resources low area-level socioeconomic status communities possess.

## Data Availability

No new data were generated or analyzed for this editorial.
